# Pediatric dentists' participation in advocacy and acceptance of new medicaid children in clinical practice

**DOI:** 10.3389/froh.2022.923124

**Published:** 2022-08-03

**Authors:** Arjun Deo Singh, Jenna Lavin, Caitlin DiStefano, Eun Chon, Stephanie Weinstein, Samantha Slutsky, Vinodh Bhoopathi

**Affiliations:** ^1^ACE Dental Clinic, Bellmead, TX, United States; ^2^Family Health Centers of Southwest Florida, Port Charlotte, FL, United States; ^3^St. Christopher's Hospital for Children, Philadelphia, PA, United States; ^4^Mount Vernon Modern Dentistry, Alexandria, VA, United States; ^5^Philadelphia V.A. Medical Center, Wynnewood, PA, United States; ^6^A1 Dental Clinic, Philadelphia, PA, United States; ^7^Section of Public and Population Health, University of California at Los Angeles School of Dentistry, Los Angeles, CA, United States

**Keywords:** advocacy, Medicaid, pediatric dentist, social responsibility, medicaid acceptability

## Abstract

**Introduction:**

Advocacy involves promoting a noble cause or voicing on behalf of a program, policy, or population group. Previous literature shows that dentists who provide services to Medicaid-enrolled, underserved, and vulnerable children are more compassionate than those who do not.

**Aim:**

To explore the association between pediatric dentists' (PDs) participation in various advocacy-related activities (ARAs) and their monthly acceptance of new Medicaid-enrolled children in their clinical practice to provide dental care services.

**Methods:**

A 14-item pilot-tested survey was created on the SurveyMonkey^®^ online platform and emailed to 5591 PDs, active American Academy of Pediatric Dentistry members. Data from 789 PD respondents were analyzed. Frequencies, percentages, means, and standard deviations were used to describe the sample. Independent *t*-tests and chi-square tests assessed the differences between PDs accepting new Medicaid-enrolled children in their clinical practice every month vs. PDs who did not. A multivariable adjusted logistic regression model determined if there was an association between PDs' participation in ARAs and their acceptance of new Medicaid-enrolled children in their clinical practice, controlling for other independent variables.

**Results:**

The mean number of different ARAs performed by PDs was 2.2 ± 1.8. Approximately 65% reported that they accepted new Medicaid-enrolled children every month in their dental clinic to provide dental care services. The multivariable logistic regression model showed that the odds of a PD accepting new Medicaid-enrolled children every month increased by 13% for each additional unit increase in ARA completed, with other variables being held constant (Odds ratio: 1.13, 95% CI: 1.03–1.25, *p* = 0.01).

**Conclusion:**

PDs who performed more ARAs had greater odds of accepting new Medicaid-enrolled children into their dental practice every month. Education and training in oral health advocacy during dental education for dental students may promote performing ARAs and providing dental care services to Medicaid patients after graduation.

## Introduction

Medicaid is one of the most extensive health insurance programs in the U.S., funded by federal and state governments to provide free or low-cost healthcare coverage for those with limited incomes, pregnant women, and people with disabilities [1, 2]. In January 2021, ~73.8 million individuals were enrolled in the Medicaid program [3]. However, participation rates of general dentists in the Medicaid or the Children Health Insurance Program (CHIP) have always been low, with only 43% participating in these programs [4]. The low dentist acceptance rate of Medicaid patients is a significant problem, leading to access to dental care issues [5]. Some of the difficulties that Medicaid patients report in accessing dental health care services include difficulty finding dentists who accept Medicaid patients, excessive wait times, rude behavior by dental staff and dentists, and discrimination by dentists because they are enrolled in Medicaid [5]. On the other hand, some studies examining barriers to dentists accepting Medicaid patients found that the most common obstacles are complicated paperwork, frequent regulations changes, slow reimbursement, and fingerprint requirements [5–7]. Low reimbursement rates and Medicaid patients missing their dental appointments have also been cited as barriers for dentists to participate in Medicaid [8, 9].

Despite Medicaid's administrative drawbacks, many dentists still participate in this program and provide dental care services to those in need. A few earlier studies described the characteristics of dentists who accept and do not accept Medicaid patients. Studies show that non-Caucasian and ethnic minority dentists served more Medicaid patients than other groups [10, 11]. Dentists in group practices accepted more Medicaid patients than solo practitioners [10], while pediatric dentists provided more care to Medicaid patients than general dentists [12, 13]. Previous studies show that male dentists and those working in non-metropolitan areas accepted more Medicaid patients than their counterparts [13–15].

A study of Iowa dentists showed that, in general, dentists who accepted Medicaid-enrolled patients had significantly higher altruistic attitudes compared to those who did not [14]. A recent study found that pediatric dentists who took new Medicaid-enrolled children every month were significantly more likely to report a willingness to advocate for community water fluoridation [16]. However, this study did not assess their participation in an activity advocating for water fluoridation or other advocacy-related activities (ARAs).

Public health advocacy involves speaking out selflessly on behalf of a program or a population and actively promoting a cause or principle [17]. A health care professional can participate in two broad advocacy-related activities (ARAs). First, as an “agent” by helping individual patients navigate and utilize health care services. Second, as an “activist” by helping advance the health of the communities and populations [18]. To be an advocate, one should be compassionate, and socially responsible. Likewise, dentists who are willing to provide services to new Medicaid-enrolled children every month, despite knowing the financial and administrative setbacks of the Medicaid program, can be considered compassionate and socially responsible. Therefore, we explored the relationship between participating in different ARAs and accepting new Medicaid-enrolled children in clinical practice to provide dental services. We hypothesized that Pediatric Dentists (PDs) participating in various ARAs would be at higher odds of accepting new Medicaid-enrolled children into their dental practice.

## Methods

### Survey instrument

This cross-sectional study was conducted using a 14-item pilot-tested survey instrument. The questions were adapted from previously conducted research studies and published advocacy toolkits [19–23]. The survey collected the following data but was not limited to: age, gender (Male/Female), year graduated from the pediatric dental residency program, type of practice setting in which PDs worked primarily (Solo practice, single group specialty, group multi-specialty, county health department, community health center, Federally Qualified Health Center (FQHCs), State or federal correctional facility clinic, other state government clinical setting, military facility clinic, Veteran Affairs (VA) clinic, academic institution, Indian Health Service), location of the primary practice (Rural/Sub-urban/Urban/Inner City), and previous training in oral health advocacy during dental education (Yes, during residency training/Yes, during predoctoral training/Yes, during both residency and predoctoral training/No training). Using a check box option, dentists were asked to select all ARAs they participated in after graduating from their pediatric dental residency training. The list of 8 ARAs include: (1) Write to an editor of a leading newspaper urging them to report on particular oral health or overall health issue (2) Communicate on Facebook or Twitter or by E-mail to promote better health, (3) Discuss a significant health issue during a town hall meeting or a public forum, (4) Participate in a community rally for a great cause, (5) Advocate for community water fluoridation at city/ local water board meetings, (6) Work with a coalition or group to improve the health of the community, (7) Donated dental services on Give Kids a Smile Day, at free clinics or through community outreach, and (8) Participation in other advocacy efforts (open-ended response).

### Data collection

The survey was adapted into an online format using the Survey Monkey^®^ (www.surveymonkey.com) platform. The online survey was administered to 5,591 pediatric dentists (PD) who were members of the American Academy of Pediatric Dentistry (AAPD) practicing in the US. The AAPD provided the list of active PD members and their emails (*n* = 5,591). PDs were given a choice to opt-out after being introduced to the purpose of the study. After the initial email message with the survey link was sent to the PDs in January 2019, three additional reminders at 2-week intervals were sent to improve the response rate. The survey was open for completion until the end of March 2019.

### Statistical data management

Several new variables were created. A “years since graduation” variable was calculated by subtracting the year the PD reported graduating from pediatric dental residency from the year the survey was conducted (2019). A new variable (Practice Location) reflecting the primary practice location was dichotomized into rural/suburban areas or urban/inner-city practices. Dental “practice settings” were recategorized into safety-net settings (county health department, community health center, FQHCs, state or federal correctional facility clinic, other state government clinical setting, military facility clinic, Veteran Affairs clinic, Academic Institution, and Indian Health Service) vs. non-safety net settings (solo practice, group single-specialty practice, and group multi-specialty practice). A new variable, “prior training in oral health advocacy during dental education” (Yes/No), was created. Those who had oral health advocacy training during pediatric dental residency, predoctoral training, or residency and predoctoral training were considered to have received training during dental education. In contrast, those without exposure were considered not to have been trained in advocacy during dental education.

#### Outcome variable

Accepting new Medicaid-enrolled children every month by PDs into their primary clinical dental practice (Yes/No) was used as the primary outcome variable.

#### Primary independent variable

A new cumulative score variable was created to determine the mean number of ARAs that PDs participated in after graduating from the pediatric dental residency program by summing all the positive responses to the 8 ARA statements (cumulative score range 0 to 8). A higher cumulative score indicates that PDs participated in more ARAs.

### Statistical analysis

Descriptive analysis was performed to understand the characteristics of the study sample. Frequency, means, standard deviations (SD), and proportions were derived. Chi-square tests and Student *t*-tests were performed to compare dentists who accepted new Medicaid-enrolled children every month to those who did not. Multivariable logistic regression model determining the association between participating in various ARAs (primary independent variable) and acceptance of Medicaid-enrolled children every month (outcome variable) by PDs was conducted, controlling for the following variables: age, gender, years since graduation, practice location, practice setting, and prior advocacy training during dental education.

## Results

Out of 5,591 PDs to whom the online surveys were sent, 123 emails were not deliverable. In addition, 328 PDs opted out of the study. The total adjusted response rate was 14.4%, with 789 PDs responding to the survey. The mean age of the participants was 45.1 ± 12.8 years, with the mean years since graduation from pediatric dental residency training of 15± 13.2 years. Most responding dentists were females (57%), practicing in suburban locations (55%) and in group single-specialty settings (40%). Approximately 65% reported accepting new Medicaid-enrolled children monthly. Most PDs answered that they were not previously trained in oral health advocacy during dental education (59%) (Table 1).

**Table 1:** General characteristics of responding pediatric dentists (*n* = 789).

**Characteristics**	**Frequency^a^**	**Percentage** **(%)**
**Gender**		
Male	339	43.0
Female	449	57.0
**Practice location**		
Rural	79	10.0
Suburban	433	54.9
Urban (not inner city)	183	23.2
Inner city	68	8.6
I do not have dental practice	26	3.3
**Practice setting** ^***b***^		
Solo practice	226	28.5
Group single specialty	319	40.3
Group multi specialty	183	23.2
County health department	3	0.4
Community health center	42	5.3
Federally qualified health center	39	4.9
State or federal correctional facility clinic	1	0.1
Other state government clinical setting	3	0.4
Military facility clinic	9	1.1
Veteran affairs clinic	0	0
Academic institution	130	16.4
Indian health service	11	1.4
**Previously trained in oral health advocacy**		
Yes, only in pre-doctoral dental program	122	17.6
Yes, only in pediatric residency program	117	16.9
Yes, in both pre-doctoral and pediatric residency program	45	6.5
No	408	59.0
**Accepting new medicaid-enrolled children every month in clinical practice**		
Yes	515	65.3
No	274	34.7

^a^Not all subgroups add to total sample due to missing values. ^b^Participants can pick more than one practice setting.

**Figure 1:**
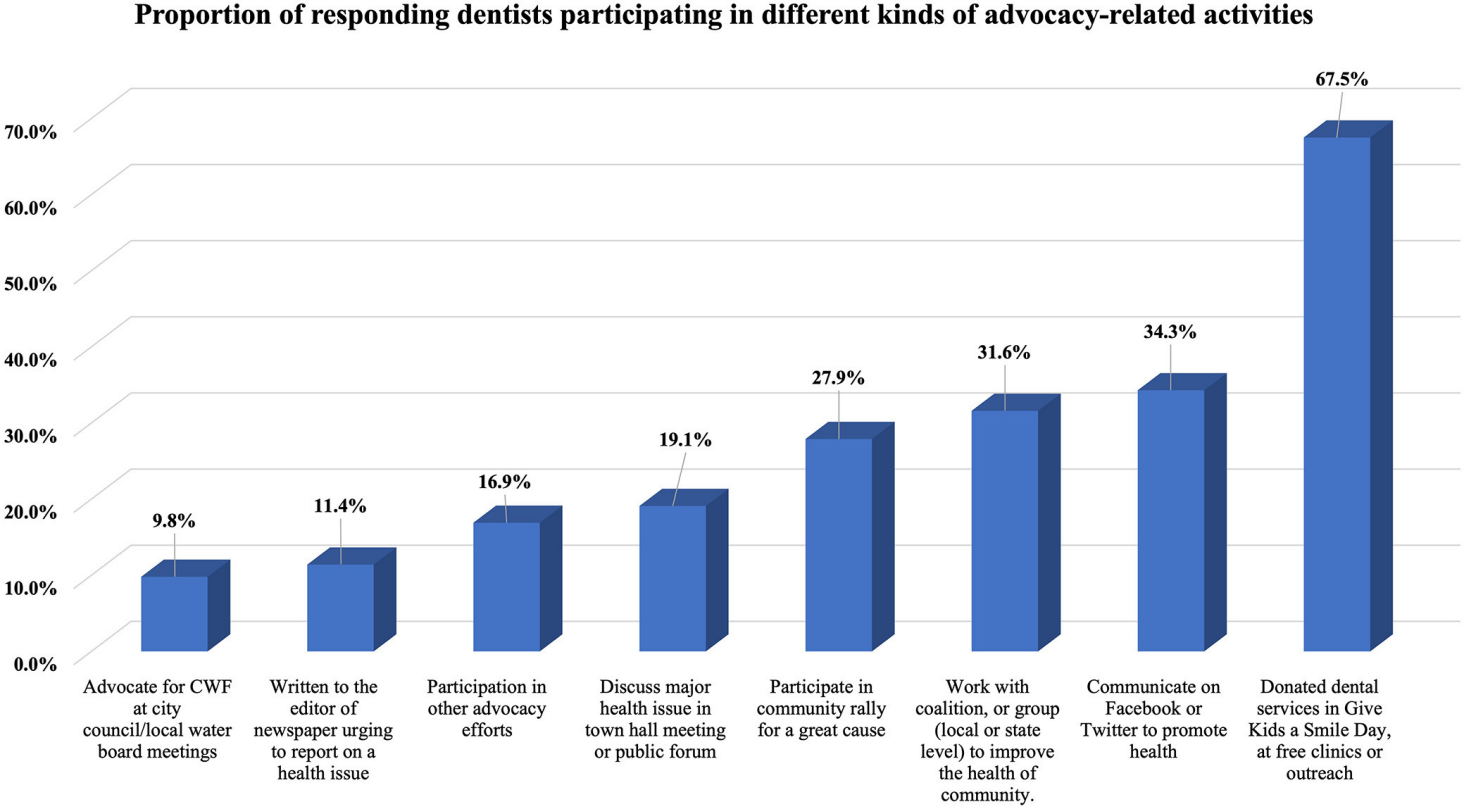
Participation in different kinds of advocacy-related activities.

The mean number of ARAs participated by PDs since graduating from a pediatric dental residency program was 2.2 ± 1.8, with Figure 1 demonstrating the distribution of ARAs. The most frequently practiced ARA was donating dental services in Give Kids a Smile day, at free clinics or through community outreach (64.5%), Facebook/Twitter communication to promote good health (34.3%), and coalition collaboration to improve community health (31.6%). The least performed activities were writing newspaper editorials/letters about oral health or overall health issue (11.4%) and community water fluoridation advocacy at city council/local water board meetings (9.8%). Nearly 17% indicated that they participated in other types of ARAs or in events representing advocacy, which include (but are not limited to): AAPD advocacy or lobby day, dental legislative day, visiting capitol hill to meet with legislators, testifying before a state legislative committee, non-profit fundraising, and membership on state advisory committees, etc.

Bivariate analysis showed no differences between the PDs who accepted and did not accept new Medicaid-enrolled children every month by age and gender (Table 2). A significant difference between those who accepted vs. not accepted new Medicaid-enrolled children every month was observed by practice location (*p* < 0.0001), practice setting (*p* = 0.001), and prior training in advocacy during dental education (*p* < 0.0001). Statistically significant differences by years since graduation were observed between dentists who accepted and did not accept new Medicaid-enrolled children every month (*p* = 0.02). PDs who accepted new Medicaid-enrolled children every month reported participating in a higher number of ARAs after graduating from the pediatric dental residency program than PDs who did not accept new Medicaid children every month (*p* = 0.006).

**Table 2:** Comparisons of pediatric dentists who accept and do not accept new Medicaid-enrolled children every month.

**Characteristic**	**Accept new medicaid children**	**Do not accept new medicaid children**	**Odds ratio**	**95% Confidence interval**	***p*-value**
**Age (Mean** **±Standard deviation)**	44.7 ± 12.9	45.7 ± 12.6	-	-	0.29
**Gender**					
Male	232 (68.4%)	107 (31.6%)	1.27	0.94–1.71	0.11
Female	283 (63.0%)	166 (37.0%)			
**Practice location**					
Inner city/Urban	198 (78.9%)	53 (21.1%)	2.30	1.62–3.27	<0.0001
Suburban/Rural	317 (61.9%)	195 (38.1%)			
**Years since graduation (Mean** **±Standard deviation)**	14.2 ± 13.3	16.5 ± 12.9	-	-	0.02
**Practice setting**					
**S**afetyNet	145 (74.7%)	49 (25.3%)	1.80	1.25–2.59	0.001
Non-SafetyNet	370 (62.2%)	225 (37.8%)			
**Trained in advocacy during dental education**					
Yes	211 (74.3%)	73 (25.7%)	1.91	1.39–2.63	<0.0001
No	304 (60.2%)	201 (39.8%)			
**Advocacy related activities performed after pediatric residency training**					
***Write to the editor of newspaper urging to report on a health issue***					
Yes	69 (76.7%)	21 (23.3%)	1.86	1.12–3.1	0.02
No	446 (63.8%)	253 (36.2%)			
***Communicate on Facebook or Twitter to promote health***					
Yes	178 (65.4%)	94 (34.6%)	1.01	0.74–1.38	0.94
No	337 (65.2%)	180 (34.8%)			
***Discuss major health issue in town hall meeting or public forum***					
Yes	106 (70.2%)	45 (29.8%)	1.32	0.90–1.94	0.16
No	409 (64.1%)	229 (35.9%)			
***Participate in community rally for a great cause***					
Yes	154 (69.7%)	67 (30.3%)	1.32	0.94–1.84	0.10
No	361 (63.6%)	207 (36.4%)			
***Advocate for CWF at city council/local water board meetings***					
Yes	55 (70.5%)	23 (29.5%)	1.30	0.78–2.17	0.31
No	460 (64.7%)	251 (35.3%)			
***Work with coalition, or group (local or state level) to improve the health of community***					
Yes	176 (70.4%)	74 (29.6%)	1.40	1.02–1.94	0.04
No	339 (62.9%)	200 (37.1%)			
***Donated services at give kids a smile day, at free clinics or through community outreach***					
Yes	354 (66.4%)	179 (33.6%)	1.17	0.85–1.59	0.33
No	161 (62.9%)	95 (37.1%)			
***Other ARAs***					
Yes	105 (77.2%)	31 (22.8%)	2.0	1.30–3.08	0.001
No	410 (62.8%)	243 (37.2%)			
***Overall ARAs (Mean** **±Standard Deviation)***	2.3 ± 1.92	1.9 ± 1.68	-	-	0.006

The multivariable logistic regression model showed that the odds of a PD accepting new Medicaid-enrolled children every month increased by 13% for each additional unit increase in ARA completed, with other variables being held constant [Odds ratio (OR): 1.13, 95% CI: 1.03–1.25, *p* = 0.01] (Table 3). PDs practicing in inner-city or urban areas (OR: 2.08, 95% CI: 1.43–3.03, *p* = 0.0001), safety net settings (OR: 2.05, 95% CI: 1.31–3.20, *p* = 0.002), and with prior advocacy training during dental education (OR: 1.61, 95% CI: 1.1–2.34, *p* = 0.01) were at higher odds of accepting new Medicaid-enrolled children every month compared to their counterparts. Age (*p* = 0.0001) and years since graduation (*p* < 0.0001) were also significant predictors.

**Table 3:** Multivariable logistic regression results showing pediatric dentists' acceptance of new Medicaid-children every month in clinical practice.

**Characteristic**	**Odds ratio**	**95% Confidence interval**	***p*-value**
**Age (Higher number)**	1.10	1.05–1.16	0.0001
**Gender**			
Male	1.40	0.98–2.00	0.06
Female	Reference		
**Practice location**			
Inner city/Urban	2.08	1.43–3.03	0.0001
Suburban/Rural	Reference		
**Years since graduation (Higher number)**	0.90	0.86–0.94	<0.0001
**Practice setting**			
**S**afetyNet	2.05	1.31–3.20	0.002
Non-SafetyNet	Reference		
**Trained in advocacy during dental education**			
Yes	1.61	1.1–2.34	0.01
No	Reference		
**Advocacy-related activities participation (Higher Number)**	1.13	1.03–1.25	0.01

## Discussion

In our study, we assessed if PDs participating in different ARAs were more inclined to take new Medicaid-enrolled children into their dental practice every month. We tested this hypothesis because advocacy is an essential attribute of the dental profession, which provides a voice to fight for a good cause for those less empowered in the community. One way the healthcare community can demonstrate social responsibility is by providing care to Medicaid recipients. Medicaid serves vulnerable populations and aims to help reduce health disparities in the community by providing care to those who cannot get it [24]. Therefore, we expected that those performing one or more ARAs would be more inclined to be accepting new Medicaid-enrolled children in their clinical practice. The results proved that our assumption was correct.

According to the American Dental Association, around 73% of PDs participated in the Medicaid and CHIP programs for child dental services in 2019 [4], while our study found a lower participation rate (65%). We specifically asked about PD's new acceptance of Medicaid-enrolled children every month to identify whether this activity was a consistent and ongoing practice, rather than reflecting Medicaid registration without providing care or providing care to a pre-existing Medicaid patient pool.

We were surprised that the responding dentists, on average, had participated in only two types of ARAs post-graduation (out of eight possible ARAs). This low level of participation could be due to PD's interest in performing a specific kind of ARA or their confidence levels in participating in a particular ARA compared to others. The most-reported ARA was providing free dental care services and communicating on social media to promote health, which was far more frequent than activities like advocating for community water fluoridation at city council meetings (the least reported ARA). PDs may find communicating with the public on social media more meaningful or easier to accomplish, especially when technology and internet use are ubiquitous. In addition, making a social media post or communication does not require much additional time or resources. On the other hand, advocating for water fluoridation in city council meetings may require different skills and more time from a pediatric dentist's everyday busy life. This reasoning is supported by a prior study assessing PDs' willingness to advocate for water fluoridation, which found that almost 13% of unwilling dentists expressed lack of time as a significant barrier [16].

Approximately 41% of the responding participants reported being trained in public health advocacy during dental school. We discovered that receiving advocacy training during dental education made PDs 65% more likely to accept new-Medicaid enrolled children every month than those without training. Previous studies show that integrating a legislative advocacy project into undergraduate or graduate-level courses positively impacts students' knowledge, values, and attitudes [25, 26] and self-efficacy skills [27]. The findings from these studies and our study indicate the importance of integrating advocacy training during dental education. Advocacy training may sensitize students and prepare them to accept and provide dental services to underserved Medicaid patients with high dental care needs. However, due to the cross-sectional nature of this data, we cannot determine if advocacy training during dental education directly impacted PD's willingness to accept new-Medicaid enrolled children. This association needs to be explored further.

Practice location and practice settings were two factors that were strongly associated with accepting new Medicaid-enrolled children by PDs. In our study, those working in urban/inner-city (metropolitan) areas were more likely to accept Medicaid children. This finding contrasts with previous studies where dentists from rural or non-metropolitan areas were more likely to take Medicaid patients [13, 28]. PDs working in safety-net settings were significantly more likely to accept new Medicaid children than those working in non-safety net environments. This is expected since serving Medicaid and uninsured patients is the sole safety-net setting mission [29]. We also found that for every unit increase in PD's age, there was a 10% increase in accepting Medicaid-enrolled children indicating that older PDs were more likely than younger PDs to accept new Medicaid-enrolled children. Again, this finding contrasts with a few other previous studies where age was not a significant predictor of Medicaid participation [13, 14].

Our study is not without limitations. First, the response rate was very low, which increased the chances of non-response bias. Though the response rate was low, the sample size was adequate for multivariable logistic regression analysis with good power. Secondly, as this survey was voluntary and was self-reported, there is a chance that PDs may have over-reported their actual level of ARA participation, leading to a possible social desirability bias. Lastly, as the nature of the study was cross-sectional, we could not determine a causal relationship between being trained in advocacy during dental school and acceptance of new Medicaid enrolled children every month. In addition, we did not inquire about the frequency with which they performed a particular ARA, which would shed more light on the global scope of PD involvement in public health advocacy.

### Conclusions

PDs who participated in more ARAs were more likely to accept new Medicaid-enrolled children every month in their clinical practice to provide dental care services. Training in oral health advocacy during dental education may enhance advocacy practices/behaviors of PDs, such as the ARAs measured in this study. Advocacy training has the potential to promote participation in ARAs after graduation, and enhance other socially responsible behaviors like accepting new Medicaid patients in clinical practice to provide dental care services.

## Data availability statement

The raw data supporting the conclusions of this article will be made available by the authors, without undue reservation.

## Ethics statement

This study was reviewed and approved as exempt by the Institution Review Board at Temple University (Protocol number: 25568). The patients/participants provided their written informed consent to participate in this study.

## Author contributions

JL, CD, EC, SW, and SS were involved in developing the survey and data collection. AS and VB was involved in analyzing the data, preparing the manuscript, and completed the first draft of the work. VB was involved in conceptualizing the project, developing the survey, study design, collecting the data, reviewing, and submitting the manuscript. All authors revised the manuscript critically for important intellectual content, reviewed the manuscript, and approved the final version to be published.

## Funding

This study was funded by the Temple University Kornberg School of Dentistry (TUKSoD), Philadelphia, PA. The study was conducted while all authors were at TUKSoD.

## Conflict of interest

The authors declare that the research was conducted in the absence of any commercial or financial relationships that could be construed as a potential conflict of interest.

## Publisher's note

All claims expressed in this article are solely those of the authors and do not necessarily represent those of their affiliated organizations, or those of the publisher, the editors and the reviewers. Any product that may be evaluated in this article, or claim that may be made by its manufacturer, is not guaranteed or endorsed by the publisher.
